# Selections

**Published:** 1886-03

**Authors:** 


### Selections.

A case of abdominal pregnancy is reported in the *Pacific Medical and Surgical Journal* by Dr. Clinton Cushing. The patient, aged 34, had given birth to one child fifteen years ago; menstruation regular until twelve months ago, when it ceased and then supervened the symptoms of pregnancy: morning sickness, enlargement of the breasts and abdomen, and, in due time, the movements of the child. Operative measures were at first postponed awaiting some verification of the diagnosis, but, as the general health of the patient began to fail rapidly, laparotomy was finally decided on.

"It was the work of but a few moments to enlarge the incision in the abdominal wall and in the sac and remove the child, which was fully developed, and about the average size of children born at full term. The child was a female, and the surface was macerated to a degree that the skin slipped off on handling.

The placenta was attached to the upper and left side of the sac very loosely; it was macerated and of a yellowish brown color, and was removed without causing any bleeding. The opening in the sac was now drawn well up to the abdominal opening and very thoroughly washed out.

The peritoneal cavity was also carefully washed out and sponged, and an examination made of all the parts.

The sac containing the child was about a quarter of an inch thick, and was developed on the left side of the uterus.

The uterus was quite small and in the right iliac region.

The opening in the sac was now united to the edge of the abdominal wound with silk sutures, a compress of absorbent cotton placed over the opening, and a bandage applied.

There was apparently no shock from the operation, for the pulse was better when she came out from under the influence of the ether than when we began.

At the end of twenty-four hours, the temperature was 100°, pulse fair, as to quality, but the nausea, which had been persistent for several days before the operation, continued; pain controlled by McMunn's elixir of opium in forty-drop doses *pro re nata.*

She died from exhaustion sixty hours after the operation."

Dr. Cushing concludes: "From Marion Sims' observation, we learn that the spermatozoa travel at the rate of three and a half inches in four hours, and, while we do not know how long they retain their vitality and motion in the cavity of the body of the female, we know that they have been found in motion on the surface of the ovary seven days after sexual connection. From recent observations made by Lawson Tait in opening the abdomen of women during ovulation, it has been found that while the funnel-shaped outer extremity of the Fallopian tube was applied closely to the surface of the ovary, it was not always applied to that particular part of the ovary from which the egg was escaping. As a consequence, the ovum, when extruded, falls into the peritoneal cavity instead of passing into the Fallopian tube, and thus an abdominal pregnancy might result.

Parry, in his excellent work on extra-uterine pregnancy, calls attention to one symptom which is invariably present in addition to the various other symptoms of pregnancy, and which was a marked symptom of this case, and that is, attacks of severe pain in the region of the uterus and ovaries.

No treatment has proved so successful in the early months of extra-uterine pregnancy as the destruction of the life of the fœtus by passing a Faradic current of electricity through the sac for fifteen minutes every third day, until the cessation of nausea, the lessening of the size of the breasts and of the tumor, give reason to believe that the fœtal life is destroyed. The sac then contracts, its contents become encysted and absorbed, and no further trouble ensues. The circuit is made by placing one sponge electrode in the roof of the vagina, the other on the surface of the abdomen.

If the child be fully developed, as in this case, the abdomen should be opened, the child removed, the placenta left in situ until it has been loosened by suppuration, and the cut edges of the sac stitched to the abdominal wound. Either this must be done or the chances taken between a mummification of the fœtus in its sac, and its escape piecemeal through the abdominal wall, the vagina, the bladder, or the intestines."

